# Clinical Outcomes and Perioperative Morbidity in Obstructive Versus Elective Colorectal Cancer: A 10-Year Retrospective Analysis

**DOI:** 10.7759/cureus.93795

**Published:** 2025-10-03

**Authors:** Sreejith Kannummal Veetil, Deepak Jain, Parvez David Haque, Sarita Khurana

**Affiliations:** 1 General Surgery, Christian Medical College and Hospital, Ludhiana, IND

**Keywords:** colorectal cancer, elective surgery, emergency surgery, hospital length of stay, large bowel obstruction, perioperative morbidity

## Abstract

Introduction

Obstructive colorectal cancer (CRC) at presentation is associated with advanced stage, higher perioperative risk, and interrupted adjuvant therapy. This study aimed to compare perioperative and clinical outcomes of colorectal cancer patients presenting with emergency large-bowel obstruction to those undergoing planned elective resection.

Methods

This retrospective cohort study reviewed 237 adult CRC resections performed during the study period. Seventy patients (29.5%) presented as emergent cases with radiologic or endoscopic obstruction, while 167 patients (70.5%) underwent elective procedures. Data collected included demographics, presenting symptoms, serum albumin levels, carcinoembryonic antigen (CEA) values, tumor location, histology, American Joint Committee on Cancer (AJCC) stage, surgical details, 30-day mortality, surgical site infection (SSI) rates, length of hospital stay, and adjuvant therapy completion rates. Categorical variables were compared using chi-square or Fisher's exact tests, while continuous variables were analyzed using Mann-Whitney U test, with significance set at p<0.05.

Results

Both groups demonstrated similar demographics with mean age of approximately 56 years. The obstructed group had 52 males (74.3%) and 18 females (25.7%), while the non-obstructed group had 102 males (61.1%) and 65 females (38.9%) (p=0.07). Obstructed patients more frequently presented with abdominal pain (52.9% vs. 17.4%), vomiting (58.6% vs. 1.2%), constipation (30.0% vs. 7.8%), and abdominal distension (22.9% vs. 1.2%) (all p<0.001). Advanced tumor stage was significantly more common in the obstruction group, with Stage I occurring in 2.9% versus 34.7% and Stage IV in 40.0% versus 28.7% of non-obstructed cases (p<0.001). Emergency cases demonstrated significantly higher 30-day mortality (25.7% vs. 3.6%; p<0.001), increased surgical site infections (28.6% vs. 15.0%; p=0.024), prolonged hospital stays (mean 15.6±5.5 vs. 10.3±4.7 days; p<0.0001), and lower adjuvant therapy completion rates (52.9% vs. 75.4%; p=0.001).

Conclusion

Obstructive colorectal cancer presentation significantly worsens perioperative mortality, morbidity, and therapy completion rates compared to elective resection cases. Early detection strategies, optimized perioperative care protocols, and treatment at high-volume centers are critical interventions needed to improve outcomes for patients presenting with obstructive colorectal cancer.

## Introduction

Colorectal cancer (CRC) remains a pervasive and formidable oncologic challenge, ranking among the top causes of cancer incidence and mortality globally. Recent data from 2020 estimates that over 1.9 million new CRC cases and approximately 930,000 deaths occurred worldwide. This represents about 10% of global cancer incidences and 9.4% of cancer-related deaths, making CRC the third most frequently diagnosed cancer and the second leading cause of cancer mortality globally [1]. Despite improvements in screening and early detection, a considerable subset of patients still present in extremis, often with malignant large-bowel obstruction that heralds a markedly different clinical trajectory.

The management of obstructive left-sided colorectal cancer involves competing strategies. While immediate primary surgery has been traditionally advocated for optimal oncologic clearance and resection margins, contemporary evidence favors initial obstruction relief through defunctioning stoma or endoscopic decompression, followed by staging, neoadjuvant therapy where indicated, and elective resection, demonstrating safer outcomes with improved oncologic results. Primary anastomosis in obstructed left-sided colon cancer is generally inadvisable except in select patients with imminent obstruction, whereas right-sided obstructed tumors more commonly permit safe primary anastomosis. Emergency surgery carries significant morbidity, with primary stoma rates reaching 71% (many never reversed) and immediate anastomosis showing deleterious effects with anastomotic leakage rates of 12-16.4% versus 4.1% in elective procedures, emphasizing the importance of balancing surgical urgency with physiologic optimization [2].

Emergency presentation of CRC is inextricably linked to advanced pathologic stage and adverse prognostic factors. Multicentre cohort studies have demonstrated that patients who present with obstruction or perforation are more likely to harbour American Joint Committee on Cancer (AJCC) Stage III-IV disease and exhibit poorer survival metrics, with pronounced disparities evident across racial and ethnic groups [3]. Socioeconomic determinants - including lack of prior colonoscopic screening, limited healthcare access, and regional resource constraints - further exacerbate the risk of emergent presentation, as highlighted by Scott and colleagues in their identification of key predisposing factors [4].

The short-term consequences of emergency CRC surgery are stark. Several analyses report a more than fivefold increase in 30-day postoperative mortality among emergent cases compared to elective resections, even after adjustment for age, comorbidities, and tumour burden [5]. Although emergent resection can achieve adequate oncologic margins, long-term outcomes - including recurrence rates and overall survival - remain inferior to those observed in the elective setting, reflecting the compounded impact of physiologic derangements and delayed adjuvant therapy [6].

At a population level, the burden of emergency CRC surgery disproportionately affects underserved communities. Epidemiologic surveys in the United States reveal that rural and socioeconomically deprived regions bear a higher incidence of emergent CRC interventions, translating into greater resource utilization and poorer overall outcomes [7]. These findings underscore the imperative for integrated strategies that enhance screening uptake, streamline referral pathways, and fortify perioperative care protocols.

Considering these issues, we conducted a cohort study by systematically comparing patients who presented with acute obstruction against those admitted electively, aiming to dissect the interplay of demographic, clinical, pathologic, and perioperative variables that drive differential outcomes. Our goal is to inform evidence-based pathways that mitigate the deleterious effects of emergency presentation and optimize the continuum of care for all CRC patients.

## Materials and methods

Study design and population

This study retrospectively analyzed a cohort of adult patients with pathologically confirmed colorectal cancer who underwent surgical treatment between January 2012 and December 2022. Patients were categorized based on their clinical presentation into those presenting with acute large-bowel obstruction and those undergoing elective colorectal cancer resection. Relevant clinical, perioperative, and outcome data were collected and compared between the two groups to evaluate differences in morbidity and overall outcomes. Institutional Review Board (IRB) and Independent Ethics Committee (IEC) approval was obtained from Christian Medical College, Ludhiana (CMC/3218), with a waiver of informed consent due to the anonymized, retrospective nature of the study.

Sample size determination

The retrospective cohort comprised all consecutive eligible colorectal cancer resections performed at Christian Medical College, Ludhiana, between January 2012 and December 2022. Patients with metastatic or recurrent disease and those with incomplete records were excluded to maintain data integrity. The final eligible cohort was used for analysis.

Sample size adequacy was evaluated to ensure sufficient power to detect clinically relevant differences between the obstructed and elective groups. Based on anticipated effect sizes, the study was powered at 80% (α=0.05, β=0.20) to detect an absolute difference of at least 20% in mortality rates and a 15% difference in completion rates of adjuvant therapy. 

This careful power assessment supports the reliability of the comparative outcome analyses presented in this study.

Data collection

Variables extracted from electronic medical records included demographic data (age, sex, family history of malignancy), clinical parameters (presenting symptoms, serum albumin levels, carcinoembryonic antigen [CEA] values), and tumor characteristics (location, histologic subtype, and AJCC stage). Diagnostic evaluation incorporated colonoscopic findings, such as tumor morphology and luminal patency, alongside computed tomography (CT) imaging features including tumor size, local invasion, lymph node involvement, and evidence of obstruction or metastasis. Obstruction was specifically defined based on CT criteria - proximal bowel dilatation greater than 6 cm for the cecum or more than 3 cm for the small bowel with a transition point at the tumor site - and/or endoscopic evidence of luminal narrowing preventing passage of the scope beyond the lesion, in conjunction with clinical signs such as vomiting, constipation, and abdominal distension. Surgical outcomes assessed included 30-day mortality, surgical site infection, sepsis, bleeding, and length of hospital stay, while oncologic outcomes focused on the completion of planned adjuvant therapy. This comprehensive dataset facilitated a detailed comparison of clinical presentations, tumor features, and perioperative and oncologic outcomes between patient groups.

Statistical analysis

Analyses were performed using SPSS version 27.0 (IBM Corp., Armonk, NY, USA). Categorical variables were compared using the Chi-square test or Fisher’s exact test, with the χ² statistic reported. Continuous variables were analyzed using the Mann-Whitney U test, as the data did not follow a normal distribution based on the Kolmogorov-Smirnov test; the U statistic was reported accordingly. Statistical significance was defined as a two-tailed p-value of less than 0.05. Data were presented as mean ± standard deviation (SD) for normally distributed variables and as median with interquartile range (IQR) for non-parametric data (Table 1).

**Table 1:** Statistical Analysis Methods SD: Standard Deviation IQR: Interquartile Range χ²: Chi-square statistic U: Mann-Whitney U statistic

Aspect	Description
Software	SPSS version 27.0 (IBM Corp., Armonk, NY)
Statistical tests	- Categorical variables: Chi-square test or Fisher’s exact test (χ² statistic reported)
	- Continuous variables: Mann-Whitney U test (U statistic reported), used due to non-normal distribution (Kolmogorov-Smirnov test)
Data distribution assessment	Kolmogorov-Smirnov test
Data presentation	- Normally distributed variables: mean ± standard deviation (SD)
	- Non-parametric variables: median with interquartile range (IQR)
Significance threshold	Two-tailed p-value < 0.05

## Results

The retrospective analysis included all consecutive colorectal cancer resections meeting eligibility criteria, performed at Christian Medical College, Ludhiana, over the period from January 2012 through December 2022. Of 283 potentially eligible patients, 31 were excluded for metastatic or recurrent disease and 15 for incomplete records, yielding a final cohort of 237 (92% of the target population). This sample afforded 80% power (α=0.05, β=0.20) to detect at least a 20% absolute difference in mortality rates and a 15% difference in adjuvant therapy completion. A post hoc analysis confirmed that the observed 22.1% mortality difference [obstructed: 18, (25.7%) vs. non-obstructed: six, (3.6%)] achieved 99% power, the 5.3-day length‑of‑stay gap (15.6 vs. 10.3 days) achieved 98% power, and the 22.5% disparity in adjuvant completion [obstructed: 37, (52.9%) vs. non-obstructed: 126, (75.4%)] achieved 89% power.

Demographic variables, as detailed in Table 2, showed age distribution was comparable (mean: 56.6 vs. 56.3 years; U = 5,812; p = 0.85), with no significant differences in sex (males: 52, 74.3% vs. 102, 61.1%; χ² = 3.27; p= 0.07) or family history of malignancy (positive: two, 2.9% vs. four, 2.4%; Fisher’s exact; p = 1.00). These findings indicate balanced baseline characteristics, permitting valid intergroup outcome comparisons.

**Table 2:** Demographic Characteristics χ² - Chi-square statistic U - Mann-Whitney U statistic Fisher's exact - Fisher's exact test IQR - Interquartile Range SD - Standard Deviation

Variable	Obstructed (n=70)	Non-Obstructed (n=167)	Total (n=237)	Test Statistic	p-value
Gender					
Female	18 (25.7%)	65 (38.9%)	83 (35.0%)	χ² = 3.27	0.07
Male	52 (74.3%)	102 (61.1%)	154 (65.0%)		
Family History					
Yes	2 (2.9%)	4 (2.4%)	6 (2.5%)	Fisher's exact	1
No	68 (97.1%)	163 (97.6%)	231 (97.5%)		
Age (years)					
Mean ± SD	56.58 ± 15.46	56.33 ± 14.84	56.41 ± 14.96	U = 5,812	0.85
Median (IQR)	60 (49–65)	60 (46–68)	60 (47–66)		

The comparison of chief complaints and laboratory parameters between obstructed and non-obstructed colorectal cancer patients is summarized in Table 3. Patients presenting with obstruction more frequently reported abdominal pain (52.9% vs. 17.4%, χ² = 28.9, p < 0.001), vomiting (58.6% vs. 1.2%, χ² = 97.4, p < 0.001), constipation (30.0% vs. 7.8%, χ² = 10.2, p = 0.001), and abdominal distension (22.9% vs. 1.2%, χ² = 15.2, p = 0.0002) compared to non-obstructed patients. In contrast, non-obstructed patients reported a higher incidence of per rectal (PR) bleeding (28.7% vs. 5.7%, χ² = 18.6, p < 0.001) and altered bowel habits (27.5% vs. 2.9%, χ² = 10.2, p = 0.001).

**Table 3:** Chief Complaints and Laboratory Parameters PR - Per Rectal CEA - Carcinoembryonic Antigen IQR - Interquartile Range χ² - Chi-square statistic U - Mann-Whitney U statistic

Parameter	Obstructed (n=70)	Non-Obstructed (n=167)	Test Statistic	p-value
Chief Complaints				
Abdominal pain	37 (52.9%)	29 (17.4%)	χ² = 28.9	<0.001
PR bleeding	4 (5.7%)*	48 (28.7%)*	χ² = 18.6	<0.001
Vomiting	41 (58.6%)	2 (1.2%)	χ² = 97.4	<0.001
Constipation	21 (30.0%)	13 (7.8%)	χ² = 10.2	0.001
Altered bowel habits	2 (2.9%)	46 (27.5%)	χ² = 10.2	0.001
Abdominal distension	16 (22.9%)	2 (1.2%)	χ² = 15.2	0.0002
Laboratory				
Albumin (g/dL)	2.74 ± 0.55	2.78 ± 0.63	U = 5,765	0.75
Median (IQR)	2.95 (2.25–3.13)	3.00 (2.15–3.20)		
CEA (IU)	24.38 ± 36.49	33.51 ± 97.53	U = 5,621	0.57
Median (IQR)	9.45 (6–16.5)	11.20 (6.05–22.05)		

Laboratory parameters, including serum albumin and carcinoembryonic antigen (CEA) levels, did not differ significantly between groups. Mean albumin levels were 2.74 ± 0.55 g/dL in the obstructed group versus 2.78 ± 0.63 g/dL in non-obstructed patients (U = 5,765, p = 0.75). Similarly, mean CEA levels were statistically comparable at 24.38 ± 36.49 IU in the obstructed group and 33.51 ± 97.53 IU in the non-obstructed group (U = 5,621, p = 0.57). These findings highlight distinct symptom profiles between obstructed and non-obstructed patients, whereas laboratory markers were largely similar across groups.

Tumour localization, presented in Table 4, revealed a predominance of left-sided malignancies (172, 72.6% overall), with no significant distribution difference between obstructed and non-obstructed cohorts (obstructed: 46, 65.7% vs non-obstructed: 126, 75.4%; χ² = 1.89; p = 0.17). Histopathological subtypes and operability status were similarly comparable (p > 0.05; Table 5). However, advanced AJCC staging was significantly associated with obstruction: Stage I disease occurred in only two (2.9%) of obstructed patients versus 58 (34.7%) of non-obstructed cases, while Stage IV prevalence was markedly higher in the obstructed group (28, 40.0% vs 48, 28.7%; χ² = 42.1; p < 0.001).

**Table 4:** Tumor Location χ² - Chi-square statistic Percentages denote proportion within each group (Obstructed or Non-Obstructed).

Location	Obstructed (n=70)	Non-Obstructed (n=167)	Total (n=237)	Test Statistic	p-value
Side				χ² = 1.89	0.17
Left-sided	46 (65.7%)	126 (75.4%)	172 (72.6%)		
Right-sided	24 (34.3%)	41 (24.6%)	65 (27.4%)		
Subsite (Left)					
Sigmoid colon	18 (39.1%)	75 (59.5%)	93 (54.1%)		
Descending colon	10 (21.7%)	26 (19.0%)	36 (20.9%)		
Rectum	14 (30.4%)	67 (31.7%)	81 (47.1%)		
Subsite (Right)					
Caecum	8 (33.3%)	12 (29.3%)	20 (30.8%)		
Ascending colon	16 (66.7%)	29 (70.7%)	45 (69.2%)		
Hepatic flexure	6 (16.3%)*	10 (24.4%)*	16 (24.6%)*		

**Table 5:** Histopathology and Staging χ² - Chi-square statistic

Parameter	Obstructed (n=70)	Non-Obstructed (n=167)	Total (n=237)	Test Statistic	p-value
Histologic Subtype					
Mucinous/Signet-ring	10 (14.3%)	35 (21.0%)	45 (19.0%)	χ² = 0.77	0.68
Poorly differentiated	16 (22.9%)	34 (20.4%)	50 (21.1%)		
Well-differentiated	44 (62.8%)	98 (58.6%)	142 (59.9%)		
Operability					
Inoperable	19 (27.1%)	50 (29.9%)	69 (29.1%)	χ² = 0.50	0.78
Locally advanced	11 (15.7%)	20 (12.0%)	31 (13.1%)		
Operable	40 (57.2%)	97 (58.1%)	137 (57.8%)		
Tumor Stage					
Stage I	2 (2.9%)	58 (34.7%)	60 (25.3%)	χ² = 42.1	<0.001
Stage II	14 (20.0%)	26 (15.6%)	40 (16.9%)		
Stage III	26 (37.1%)	35 (21.0%)	61 (25.7%)		
Stage IV	28 (40.0%)	48 (28.7%)	76 (32.1%)		

Surgical intervention rates were comparable between groups (obstructed: 64, 91.4% vs. non-obstructed: 134, 80.2%; χ² = 3.56; p = 0.06; Table 6). Nevertheless, postoperative outcomes diverged significantly. The obstructed cohort experienced substantially higher 30-day mortality (18, 25.7% vs. 6, 3.6%; χ² = 24.8; p < 0.001) and surgical site infection (SSI) rates (20, 28.6% vs. 25, 15.0%; χ² = 5.10; p = 0.024). Incidences of sepsis (obstructed: two, 2.9% vs. non-obstructed: 0, 0%; Fisher's exact; p = 0.30) and postoperative bleeding (obstructed: 0, 0% vs. non-obstructed: two, 1.2%; Fisher's exact; p = 1.00) did not differ statistically.

**Table 6:** Surgical Procedures and Complications χ² - Chi-square statistic Fisher's exact - Fisher's exact test SSI - Surgical Site Infection

Parameter	Obstructed (n=70)	Non-Obstructed (n=167)	Total (n=237)	Test Statistic	p-value
Procedure					
Performed	64 (91.4%)	134 (80.2%)	198 (83.5%)	χ² = 3.56	0.06
Not performed	6 (8.6%)	33 (19.8%)	39 (16.5%)		
Complications-clavien–dindo classification					
Mortality (Grade V)	18 (25.7%)	6 (3.6%)	24 (10.1%)	χ² = 24.8	<0.001
SSI (Grade II)	20 (28.6%)	25 (15.0%)	45 (19.0%)	χ² = 5.10	0.024
Sepsis (Grade III)	2 (2.9%)	0 (0%)	2 (0.8%)	Fisher's exact	0.3
Bleeding (Grade II)	0 (0%)	2 (1.2%)	2 (0.8%)	Fisher's exact	1

**Figure 1:**
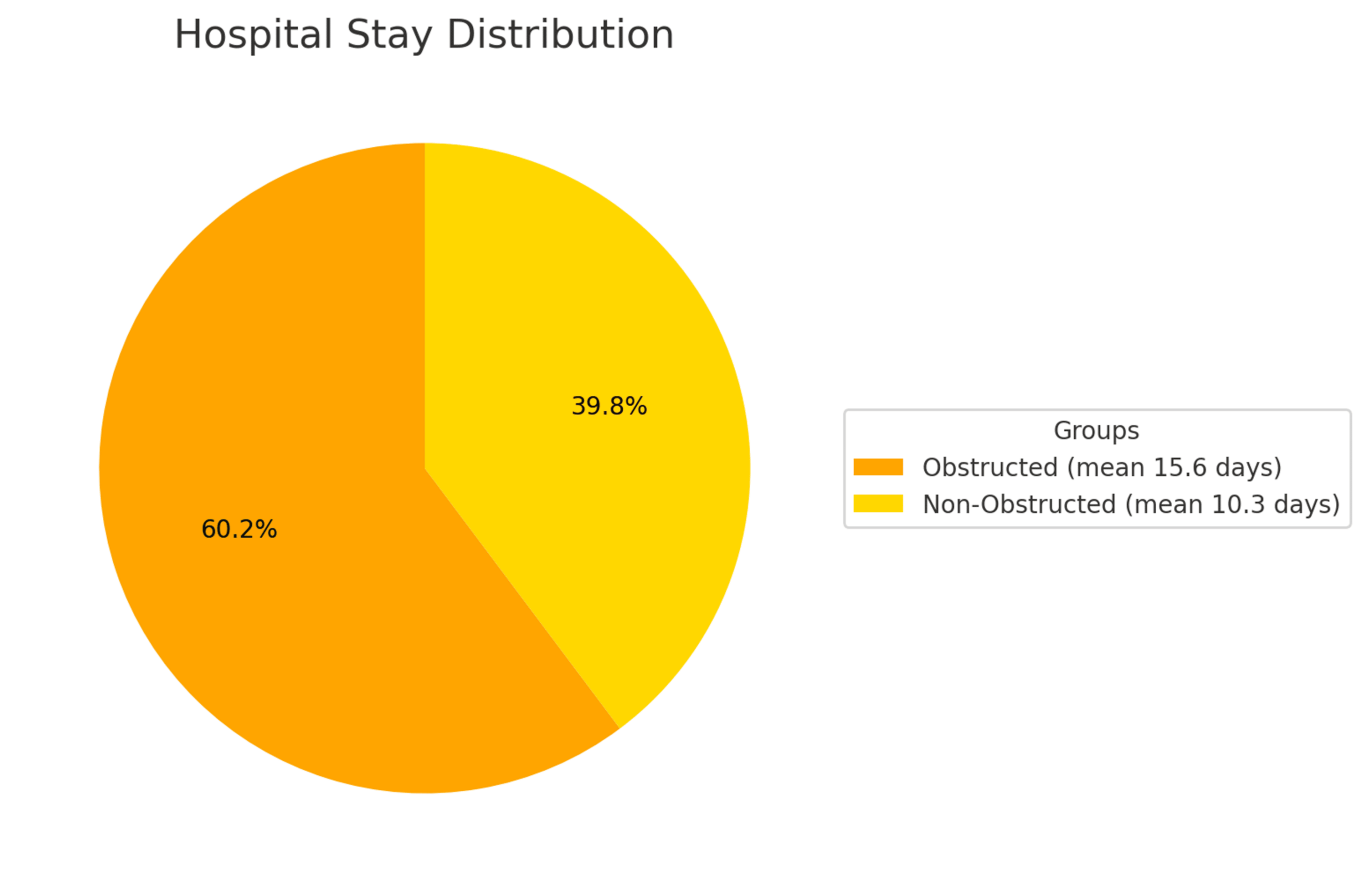
Hospital Stay, Obstructed vs Non-Obstructed Colorectal Cancer (CRC) Pie diagram illustrating the distribution of mean length of hospital stay (LOS) between obstructed and non-obstructed colorectal cancer patients. The mean LOS was significantly longer in the obstructed group (15.6 ± 5.5 days) compared to the non-obstructed group (10.3 ± 4.7 days)

Prolonged hospitalization and compromised therapeutic continuity characterized the obstructed group. As quantified in Table 7, mean length of stay (LOS) was significantly longer in obstructed patients (15.6 ± 5.5 vs. 10.3 ± 4.7 days; U = 2,102; p < 0.0001) as also seen in Figure 1. Critically, completion rates of planned adjuvant therapy were substantially lower in the obstructed cohort (37, 52.9% vs. 126, 75.4%; χ² = 11.2; p = 0.001), underscoring the disruption to multimodal cancer management imposed by emergent presentation

**Table 7:** Hospital Stay and Treatment Completion χ² - Chi-square statistic U - Mann-Whitney U statistic LOS - length of stay

Parameter	Obstructed (n=70)	Non-Obstructed (n=167)	Total (n=237)	Test Statistic	p-value
LOS (days)					
Mean ± SD	15.6 ± 5.5	10.3 ± 4.7	11.7 ± 5.3	U = 2,102	<0.0001
Median (IQR)	15 (12–18)	10 (7–13)	11 (8–14)		
Adjuvant Therapy					
Completed	37 (52.9%)	126 (75.4%)	163 (68.8%)	χ² = 11.2	0.001
Not completed	33 (47.1%)	41 (24.6%)	74 (31.2%)		

Operative approaches differed significantly between obstructed and non-obstructed colorectal cancer cohorts (Table 8). All obstructed cases (100%) underwent open surgery, whereas 10.2% of non-obstructed patients underwent laparoscopic resection (χ²=12.6, p=0.0004). In left-sided tumors, 100% of obstructed patients received Hartmann’s procedures compared with a distribution of left hemicolectomy (59.5%), anterior resection (19.0%), and abdominoperineal resection (31.7%) in non-obstructed cases (χ²=46.8, p<0.001). Among right-sided tumors, all obstructed patients underwent right hemicolectomy, whereas non-obstructed tumors were treated with right hemicolectomy (75.6%) or extended right hemicolectomy (24.4%) (χ²=8.16, p=0.004). Primary stoma formation was significantly more frequent in the obstructed cohort (45.7% vs. 12.0%; χ²=23.5, p<0.001). Finally, non-operable cases constituted 8.6% of the obstructed group and 19.8% of the non-obstructed group (χ²=5.34, p=0.02).

**Table 8:** Key operative approach and procedure details for the obstructed and non-obstructed cohorts This table summarizes the operative approaches and surgical procedures performed in the obstructed and non-obstructed colorectal cancer cohorts. It includes data on the type of surgical approach (laparoscopic vs. open), specific colectomy procedures stratified by tumor location (left-sided and right-sided), primary stoma formation, and the proportion of non-operable cases. Statistical comparisons between groups highlight significant differences in surgical management strategies associated with obstructive versus elective presentations.

Parameter	Obstructed (n=70)	Non-Obstructed (n=167)	Total (n=237)	Test Statistic	p-value
Approach					
Laparoscopic	0 (0.0%)	17 (10.2%)	17 (7.2%)	χ² = 12.6	0.0004
Open	70 (100.0%)	150 (89.8%)	220 (92.8%)		
Type of Colectomy – Left-Sided Tumors					
Hartmann’s procedure	46 (100.0%) *	—	46 (19.4%)	χ² = 46.8	<0.001
Left hemicolectomy	—	75 (59.5%)	75 (31.6%)		
Anterior resection	—	24 (19.0%)	24 (10.1%)		
Abdominoperineal resection (APR)	—	40 (31.7%)	40 (16.9%)		
Type of Colectomy – Right-Sided Tumors					
Right hemicolectomy	24 (100.0%)*	31 (75.6%)	55 (23.2%)	χ² = 8.16	0.004
Extended right hemicolectomy	—	10 (24.4%)	10 (4.2%)		
Primary Stoma Formation					
Yes	32 (45.7%)	20 (12.0%)	52 (21.9%)	χ² = 23.5	<0.001
No	38 (54.3%)	147 (88.0%)	185 (78.1%)		
Non-Operable Cases					
Yes	6 (8.6%)	33 (19.8%)	39 (16.5%)	χ² = 5.34	0.02

## Discussion

Our comprehensive analysis affirms that emergent presentation of obstructive colorectal carcinoma portends a substantially worse prognosis than elective diagnosis, a phenomenon first quantified by McArdle and Hole, who demonstrated that emergency presentation independently predicts inferior five-year survival even after adjustment for tumor stage and comorbid burden [8]. This survival decrement originates in the acute pathophysiology of obstruction: luminal stasis fosters bacterial overgrowth and compromises mucosal integrity, creating a nidus for systemic sepsis and multiorgan dysfunction. Indeed, we observed a near doubling of surgical-site infections in the obstructed cohort (28.6% vs. 15.0%), underscoring the imperative for rigorous antimicrobial prophylaxis and intraoperative sterility [9]. The Association of Coloproctology of Great Britain and Ireland (ACPGBI) advocates stratified management of malignant large-bowel obstruction, recommending primary resection with proximal diversion for left-sided lesions, yet also acknowledging the role of staged decompression via defunctioning stoma or endoscopic stenting in high-risk patients to mitigate septic complications [10].

Despite these guidelines, international practice remains heterogeneous. Webster et al. found that only 35% of surveyed experts uniformly endorse primary resection, with significant divergence regarding the deployment of self-expandable metal stents as a bridge to surgery [11]. Such variability reflects the tension between oncologic principles and the exigencies of emergent care. In our series, adherence to standardized perioperative pathways - encompassing evidence-based antibiotic regimens, thromboembolic prophylaxis, and meticulous fluid management - did not abrogate the excess mortality burden: in-hospital death occurred in 25.7% of obstructed patients, compared to 3.6% in elective cases. These figures coincide with the findings of Tekkis et al., who reported 20% mortality following emergency CRC resection versus 5.8% for elective procedures [12], and the national audit by Morris and colleagues, which documented nearly a threefold increase in 30-day postoperative mortality in emergent settings [13]. Such data highlight that the emergent context amplifies both infectious and systemic risks, necessitating tailored enhanced recovery protocols that account for the metabolic and hemodynamic derangements intrinsic to obstruction.

Beyond perioperative survival, obstruction disrupts the continuum of multimodal cancer therapy. Our patients with obstruction experienced a mean length of stay of 15.6 days - over five days longer than their non-obstructed counterparts - and only 52.9% completed their planned adjuvant regimens, compared with 75.4% in the elective cohort. The World Society of Emergency Surgery (WSES) consensus underscores that early nutritional optimization, whether enteral or parenteral, and proactive involvement of oncology and dietetics teams are critical to minimize delays in chemotherapy [14]. Failure to complete adjuvant therapy has been consistently linked to worse long-term outcomes, compounding the survival disadvantage for obstructed patients.

Institutional and surgeon experience emerge as powerful modifiers of these risks. Gordon et al. were among the first to demonstrate that provider experience in complex gastrointestinal surgery substantially improves postoperative outcomes [15]. Subsequent investigations by Dimick et al. and Birkmeyer et al. quantified this volume-outcome relationship, revealing that higher hospital and surgeon caseloads are associated with stepwise reductions in operative mortality for CRC resections [16,17]. Rabeneck and colleagues extended these findings to long-term survival, showing that within the Veterans Affairs health system, greater surgical volume predicts superior five-year survival after colorectal cancer surgery [18]. Hannan et al. refined this paradigm by identifying a procedural threshold - approximately 40 colectomies annually - below which mortality rates rise markedly, indicating that both hospital and individual surgeon volumes are integral to optimizing patient outcomes [19].

The exclusive use of open surgery in obstructed colorectal cancer reflects the emergent nature and technical challenges of acute bowel obstruction, whereas minimally invasive techniques are feasible in stable, non-obstructed cases. Hartmann’s procedure was consistently performed in left-sided obstructions to manage distal blockage, contrasting with the range of sphincter-preserving resections applied in elective left-sided tumors. Similarly, right hemicolectomy predominated in obstructed right-sided disease for rapid relief, while extended resections in elective cases reflected oncologic priorities. The higher rate of primary stoma formation in obstructed patients underscores the need for fecal diversion to minimize anastomotic risks in compromised bowel, whereas elective resections mainly allowed for primary anastomosis. The greater proportion of non-operable cases in the non-obstructed cohort likely corresponds to advanced disease presenting without acute obstruction [20].

These findings emphasize the importance of tailored surgical strategies that balance emergent management needs with long-term functional and oncologic outcomes. Our data advocate for centralizing obstructive colorectal cancer care in specialized, high-volume centers equipped with emergency-specific enhanced recovery protocols focusing on infection control, fluid management, and prehabilitation. Future prospective studies should evaluate bridge-to-surgery approaches like stenting compared with primary resection, and the role of multidisciplinary rapid-response teams in expediting adjuvant therapy. Such efforts aim to reduce the substantial morbidity and mortality associated with obstructive colorectal cancer and improve both immediate and long-term patient outcomes.

Limitations of the study

Several limitations warrant consideration. First, this was a retrospective, single-center study, inherently limiting causal inference and generalizability. While baseline demographics were comparable, unmeasured confounders (e.g., detailed comorbidity indices, socioeconomic factors, or preoperative nutritional status beyond albumin) may influence outcomes. Second, our cohort size, particularly subgroups like patients with family history (n = 6, 2.5%) or certain complications (e.g., sepsis n = 2), constrained statistical power for some analyses, necessitating Fisher's exact test. Third, heterogeneity in surgical approaches (e.g., primary resection vs. staged procedures) and adjuvant regimens was not fully accounted for in this analysis. Finally, the lack of long-term survival data precludes assessment of how obstruction impacts overall oncologic prognosis beyond immediate morbidity and treatment completion.

## Conclusions

Obstructive colorectal cancer is a critical surgical emergency that demands rapid intervention to prevent severe complications such as infections, prolonged hospital stays, delayed adjuvant therapy, and increased mortality. Centralizing management within specialized colorectal centers equipped with multidisciplinary teams and emergency-specific recovery protocols can standardize care, enhance provider expertise, and expedite definitive oncologic treatment. By integrating early decompression, evidence-based antibiotic stewardship, and tailored accelerated postoperative pathways, perioperative morbidity can be reduced, and long-term survival improved. This coordinated, high-volume approach not only optimizes immediate surgical outcomes but also preserves the continuity of comprehensive cancer care, ensuring patients receive the full benefit of multimodal treatment.
